# Establishment and Preliminary Application of a Multiplex TaqMan Real-Time Fluorescence Quantitative PCR Assay for the Detection of *Pneumocystis* Species

**DOI:** 10.3390/microorganisms14061308

**Published:** 2026-06-11

**Authors:** Qiuyang Sun, Yuanzhi Xie, Yufang Feng, Qiang Gao, Rui Fu, Jin Xing

**Affiliations:** Institute of Laboratory Animal Resources (National Rodent Experimental Animal Resource Bank), National Institutes for Food and Drug Control, Beijing 102629, China; qiyang07@outlook.com (Q.S.); xieyuanzhi@nifdc.org.cn (Y.X.); fengyufang@nifdc.org.cn (Y.F.); gaoqiang@nifdc.org.cn (Q.G.); furui78@nifdc.org.cn (R.F.)

**Keywords:** *Pneumocystis murina*, *Pneumocystis carinii*, *Pneumocystis jirovecii*, molecular diagnosis, laboratory animal quality control, real-time fluorescence quantitative PCR (qPCR)

## Abstract

*Pneumocystis* is an opportunistic fungal pathogen that causes severe *Pneumocystis pneumonia* (PCP) in immunocompromised individuals and laboratory animals. Three host-specific species—*Pneumocystis murina* (*P. murina*), *Pneumocystis carinii* (*P. carinii*), and *Pneumocystis jirovecii* (*P. jirovecii*)—are closely associated with infections in humans and laboratory animals. However, the conventional method, microscopic staining, suffers from low sensitivity, operator-dependent subjectivity, and inability to differentiate species, highlighting the urgent need for a multiplex qPCR assay. In this study, we established a multiplex qPCR method targeting the *mtLSUrRNA* gene of *P. murina*, the *TS* gene of *P. carinii*, and the *mtSSUrRNA* gene of *P. jirovecii*. Primers and probes were designed and optimized using a matrix approach. The method was systematically evaluated for sensitivity, specificity, and reproducibility using recombinant plasmid standards and laboratory animal samples. Validation was performed on 260 mouse lung samples, 30 *P. murina*-positive samples, 25 rat lung samples, 6 rat bronchoalveolar lavage fluid (BALF) samples, and 8 *P. carinii*-positive samples. Results were compared with single-plex qPCR and staining microscopy (performed on 68 mouse lung samples, 38 *Pneumocystis*-positive samples). The limits of detection (LOD) were 5 copies/μL for *P. murina*, 6 copies/μL for *P. carinii*, and 8 copies/μL for *P. jirovecii*. Standard curves showed excellent linearity (R^2^ ≥ 0.999) with amplification efficiencies of 90–110%. No non-specific reactions were observed with 22 common pathogens, and intra-/inter-group coefficients of variation (CV%) were below 1%. Moreover, interference testing revealed minimal matrix effects on the amplification system and no mutual interference among the primers and probes. The multiplex qPCR detected all 38 positive samples (100%), showing 100% concordance with single-plex qPCR, whereas Giemsa staining detected none (0%) and toluidine blue staining only 60% (3/5) of the tested positives, suggesting that the multiplex qPCR achieved higher detection rates than staining microscopy. In conclusion, this novel multiplex qPCR method offers high sensitivity, specificity, and reproducibility, providing a sensitive and specific tool for laboratory animal health monitoring and epidemiological surveillance. Its clinical application for human PCP diagnosis requires further validation with authentic human specimens.

## 1. Introduction

*Pneumocystis* (PC) is a genus of obligate opportunistic fungal pathogens that exhibit strict host specificity in mammals. Three species are particularly relevant to humans and laboratory animals: *Pneumocystis murina* (*P. murina*, mice), *Pneumocystis carinii* (*P. carinii*, rats), and *Pneumocystis jirovecii* (*P. jirovecii*, humans) [1,2].

*Pneumocystis* has two developmental stages, the trophozoite and the cyst, the latter of which contains 2–8 spores and is the primary mode of transmission [3]. The organism cannot be cultured in vitro; limited proliferation is possible only in vivo [4]. Transmission occurs via airborne droplets or aerosols, though the exact mechanism in humans remains unclear [5]. The fungus can persist latently in the respiratory tract, and when CD4^+^ T-cell immunity is compromised, it causes severe *Pneumocystis pneumonia* (PCP) [6].

PCP is a leading opportunistic infection in immunocompromised individuals, including HIV/AIDS patients, transplant recipients, and those on immunosuppressive therapy [7,8,9]. Its incidence is rising, posing a public health challenge. In laboratory animals, immunodeficient mice (SCID, nude), immunosuppressed animals, and humanized mouse models are highly susceptible to spontaneous PCP [10]. The infection often subclinical, can interfere with tumor models, immunological studies, and drug evaluations, wasting resources and reducing data reliability [11,12,13,14,15,16]. Moreover, humanized mice are at risk of cross-infection between rodent-derived (*P. murina*) and potentially human-derived (*P. jirovecii*) species, highlighting the critical need for accurate species identification [17].

Currently, staining microscopy is a conventional method for *Pneumocystis* detection [18]; however, it suffers from low sensitivity, operator-dependent subjectivity, inability to differentiate species, and low throughput. Real-time fluorescence quantitative PCR (qPCR) offers high sensitivity and specificity, yet existing assays are mostly single-plex, targeting only one species per reaction. Consequently, no multiplex qPCR method is currently available for the simultaneous detection of *P. murina*, *P. carinii*, *P. jirovecii* and a gap that hampers efficient monitoring and diagnosis.

In this study, we developed a multiplex qPCR assay for the concurrent detection and differentiation of these three species. The method was systematically validated for sensitivity (detection limit as low as 5–8 copies/μL), specificity (no cross-reactivity with 22 common pathogens), and reproducibility (CV < 1%). Its applicability was successfully tested on a large panel of laboratory animal samples. This platform provides a rapid, reliable, and high-throughput tool for etiological diagnosis, epidemiological surveillance, and health monitoring of laboratory animals, thereby addressing a critical unmet need in both research and quality control settings.

## 2. Materials and Methods

### 2.1. Standard Strains, DNA and Test Samples

The standard strains of *P. murina* (PRA-111, ATCC, Manassas, VA, USA) and *P. carinii* (PRA-159, ATCC, Manassas, VA, USA), as well as reference DNA of *P. jirovecii* (MYA-5006SD, ATCC, Manassas, VA, USA), were preserved in our laboratory.

The samples used in this study were divided into two categories: (1) a total of 260 lung tissue samples from mice (SCID, Balb/c-nu, Balb/c, C57BL/6J, and NOD strains, Beijing, China), as well as 25 lung tissue samples and 6 bronchoalveolar lavage fluid (BALF)samples from rats (SD and Wistar strains, Beijing, China), all of which were stored in a −80 °C ultra-low temperature freezer; (2) positive mouse samples consisting of 30 lung tissue samples collected from mice (Balb/c, C57BL/6J, and Balb/c-nu strains, Beijing, China) experimentally infected with the *P. murina* standard strain, and positive rat samples consisting of 8 lung tissue samples collected from rats (Wistar strain, Beijing, China) experimentally infected with the *P. carinii* standard strain. All positive samples were stored in liquid nitrogen and used for assay validation and quality control. Prior to testing, all samples were removed from storage, thawed on ice, and pretreated accordingly. Repeated freezing and thawing was avoided.

### 2.2. Primer and Probe Design and Synthesis

Primers and probes were designed using Beacon Designer 8 based on target gene sequences, labeled with VIC/BHQ1 (*P. murina*), CY5/BHQ2 (*P. carinii*), and FAM/BHQ1 (*P. jirovecii*), and synthesized by Sangon Biotech (Shanghai, China) (Table 1).

### 2.3. Synthesis of Plasmid Standards

Standard plasmids were synthesized using pUC19 as a vector, based on the target gene sequences of *P. murina mtLSUrRNA* [19], *P. carinii TS* [20], and *P. jirovecii mtSSUrRNA* [21]. The synthesis was commissioned to Sangon Biotech (Shanghai, China). The resulting plasmids were designated as pUC19-P.mMT (carrying the *P. murina mtLSUrRNA* gene), pUC19-P.cTS (carrying the *P. carinii TS* gene), and pUC19-P.jmt (carrying the *P. jirovecii mtSSUrRNA* gene).

### 2.4. Optimization of Single-Plex qPCR Reaction System and Conditions

The total reaction volume for single-plex qPCR was 20 μL (GoTaq^®^ Probe qPCR Master Mix, Promega, Madison, WI, USA). Using the three recombinant plasmid standards as templates, optimization of the final concentrations of probes and primers was performed using the matrix method [22]. The probe concentrations tested were 50 nM, 100 nM, 200 nM, and 300 nM, and the primer concentrations tested were 50 nM, 100 nM, 150 nM, 200 nM, 250 nM, 300 nM, 400 nM, 500 nM, and 600 nM. The thermal cycling conditions were as follows: 50 °C for 2 min, 95 °C for 10 min, followed by 40 cycles of 95 °C for 15 s and annealing for 1 min at different annealing temperatures (58 °C, 59 °C, 60 °C, and 62 °C). Through these experiments, the reaction system and program that produced high fluorescence intensity, a low cycle threshold (C_T_) value, and an amplification efficiency between 90% and 110% were selected as the optimal reaction conditions.

### 2.5. Establishment of the Multiplex qPCR System

To establish a multiplex qPCR system for the simultaneous detection of the three *Pneumocystis* species, the specific probes for *P. murina*, *P. carinii*, and *P. jirovecii* were labeled with the fluorescent groups VIC, Cy5, and FAM, respectively, in accordance with the requirements for three-channel fluorescence detection (Table 1). The system was constructed using a stepwise approach. Firstly, based on the optimized single-plex qPCR conditions, the optimized primers and probes for two of the *Pneumocystis* species were combined to establish a duplex qPCR reaction system. Subsequently, the primers and probes for the third *Pneumocystis* species were added, and following further optimization of the conditions, the multiplex qPCR detection system was successfully established [23].

### 2.6. Establishment of Standard Curves

To establish standard curves for multiplex qPCR quantification, the three recombinant plasmid standards were serially diluted tenfold. Equal volumes of each dilution were then mixed to prepare a series of mixed templates, such that the final concentration of each plasmid covered six orders of magnitude, ranging from 1 × 10^3^ copies/μL to 1 × 10^8^ copies/μL [24]. Three replicate wells were set up for each concentration, and nuclease-free water was used as a negative control. Amplification was performed using the optimized multiplex qPCR system, and standard curves were plotted with the logarithm of the copy number (log_10_ copy number) on the x-axis and the C_T_ on the y-axis. Parallel single-plex qPCR standard curves were also generated under identical conditions using the same dilution series of each individual plasmid standard, with three replicates per concentration.

### 2.7. Specificity Tests

A panel of 22 non-target pathogens was selected as template DNA for detection, including 10 rodent respiratory bacteria (*Rodentibacter pneumotropicus*, *Mycoplasma pulmonis*, *Bordetella bronchiseptica*, *Pasteurella multocida*, *Streptococcus pneumoniae*, *Streptobacillus moniliformis*, *Corynebacterium kutscheri*, *Mycoplasma neurolyticum*, *Mycoplasma arthritidis*, and *Escherichia coli* [respiratory isolate]), 8 fungi (*Candida albicans*, *Saccharomyces cerevisiae*, *Microsporum canis*, *Trichophyton simiae*, *Cryptococcus neoformans*, *Trichophyton simiae* (monkey-derived), *Microsporum gypseum* and *Aspergillus niger*), and 4 intestinal bacteria (*Proteus mirabilis*, *Escherichia coli* [intestinal isolate], *Enterococcus faecalis*, and *Staphylococcus aureus*) [25,26]. Simultaneously, a mixed DNA sample of the three *Pneumocystis* species (DNA mix) was set as a positive control, and nuclease-free water was used as a negative control. Amplification was performed using the optimized multiplex qPCR system. The specificity of the multiplex qPCR detection method for *P. murina*, *P. carinii*, and *P. jirovecii* was evaluated based on the specific amplification curves and fluorescence signals.

### 2.8. Sensitivity Tests

To assess the detection sensitivity of the multiplex qPCR method, the three recombinant plasmid standards were serially diluted tenfold and then mixed to prepare templates containing each plasmid at concentrations of 100 copies/μL, 50 copies/μL, 20 copies/μL, 10 copies/μL, 8 copies/μL, 6 copies/μL, 5 copies/μL, and 1 copy/μL. The mixed plasmid standards were used as templates, and nuclease-free water was used as a negative control. Each concentration was tested in 22 parallel reactions. Amplification was performed using the established multiplex qPCR system, and the detection rate and coefficient of variation (CV%) were calculated. Based on these results, the lower limit of detection (LOD) of the method was determined. For comparison, single-plex qPCR assays for each target were performed in parallel at the same low concentrations of 10, 8, 6, 5 copies/μL, and 1 copy/μL (22 replicates each) to assess whether multiplexing affects sensitivity.

### 2.9. Repeatability Tests

To assess the reproducibility of the established multiplex qPCR method, mixed recombinant plasmid standards at concentrations of 1 × 10^3^ copies/μL, 1 × 10^5^ copies/μL, and 1 × 10^7^ copies/μL were used as assay templates, and nuclease-free water was used as a negative control. For the intra-group reproducibility test, each concentration template was analyzed in triplicate within the same batch. For the inter-group reproducibility test, the aforementioned assay procedure was repeated at irregular intervals, with each concentration similarly analyzed in triplicate. The stability and reproducibility of the method, both intra-group and inter-group, were comprehensively evaluated by calculating the CV of the C_T_ at each concentration.

### 2.10. Assessment of Extraction Efficiency and qPCR Inhibition (Spike-In Experiment)

To monitor DNA extraction efficiency and evaluate potential qPCR inhibition caused by tissue matrix (lung or BALF), a spike-in experiment was conducted using the recombinant plasmid standards [27]. Negative lung homogenates and BALF from pathogen-free animals were used as matrices. The experimental design is summarized in Table 2.

Extraction recovery rates were calculated as: (measured copy number in Group G/theoretical input copy number) × 100%. qPCR inhibition was assessed by comparing C_T_ between Group E (matrix + plasmid after extraction) and Group F (plasmid only). A recovery rate between 80% and 120% was considered acceptable. If significant inhibition or extraction loss was observed, a correction factor (CF = 100%/recovery rate) could be applied to adjust the final concentration of test samples.

### 2.11. Sample Detection

A total of 260 mouse lung samples, 30 *P. murina*-positive mouse samples, 25 rat lung samples, 6 BALF samples, and 8 *P. carinii*-positive rat samples were collected. The lung tissues were pretreated by cutting them into small pieces, placing them in grinding tubes, adding sterile PBS together with grinding beads, and mechanically grinding the mixture at 5 °C and 60 Hz for 30–60 s. BALF samples were left untreated. Subsequently, genomic DNA was extracted using a tissue extraction kit and served as a template for subsequent *Pneumocystis* multiplex qPCR and single-plex qPCR detection. To verify the reliability of the method, nuclease-free water was used as a negative control, while DNA mix, *P. murina*-positive samples, and *P. carinii*-positive samples were used as positive controls. The established multiplex qPCR and single-plex qPCR systems were used for simultaneous detection, and the results were compared with those of staining microscopy to validate the feasibility and efficacy of the developed method for sample application.

### 2.12. Sequencing Verification

To confirm the specificity of the multiplex qPCR assay for naturally amplified targets, PCR products from *P. murina*-positive mouse lung samples and *P. carinii*-positive rat lung samples were subjected to Sanger sequencing. The amplicons (140 bp for *P. murina* and 83 bp for *P. carinii*) were generated using the respective primers listed in Table 1 under the same thermal cycling conditions as the multiplex qPCR but without fluorescent probes. The PCR products were purified and sequenced bidirectionally by Sangon Biotech (Shanghai, China). The obtained sequences were aligned with reference sequences (GenBank accession numbers: NC_020332.1 (2768–5511) for *P. murina mtLSUrRNA* and XM_018371599.1 for *P. carinii TS*) using SnapGene (version 6.0.2) to verify target identity. The *P. jirovecii* target was not sequenced because it was derived from a recombinant plasmid standard with a confirmed sequence.

### 2.13. Statistical Analysis

Raw qPCR data (C_T_ and detection calls) were exported directly from the real-time qPCR instrument. All statistical analyses were performed using WPS Excel (version 12.1, Kingsoft Office). Quantitative data are expressed as mean ± standard deviation (SD). Standard curves were generated by linear regression of C_T_ against log_10_ copy numbers; correlation coefficients (R^2^) and amplification efficiencies (E = [10^(−1/slope)^ − 1] × 100%) were calculated from the regression equations. Detection rates for sensitivity tests are reported with 95% confidence intervals (CIs) calculated using the Clopper-Pearson exact method. Intra-group and inter-group repeatability were assessed by calculating coefficients of variation (CV% = SD/mean × 100) of C_T_.

## 3. Results

### 3.1. Optimization Results of the Single-Plex qPCR Reaction System and Conditions

The single-plex qPCR reaction system was optimized using the matrix method to determine the optimal reaction conditions for the three plasmid standards: pUC19-P.mMT, pUC19-P.cTS, and pUC19-P.jmt. The optimization results showed that the final concentrations were 100 nM for the probe of each target and 200 nM for the primers. Furthermore, the optimal annealing temperature was determined to be 60 °C. The finalized 20 μL single-plex reaction system consisted of 10 μL of 2× GoTaq Probe qPCR Master Mix, 2 μL of template DNA, and 1 μL of a 10 μM primer and probe mix, with the total volume adjusted to 20 μL using nuclease-free water. The reaction program included an incubation at 50 °C for 2 min, pre-denaturation at 95 °C for 10 min, followed by 40 cycles of amplification (95 °C for 15 s and 60 °C for 1 min).

### 3.2. Establishment of the Multiplex qPCR Reaction System

Based on the system and conditions optimized for single-plex qPCR, a multiplex qPCR reaction system was successfully developed, followed by system optimization and performance evaluation. The finalized 20 μL multiplex reaction system comprised 10 μL of 2× GoTaq Probe qPCR Master Mix, 2 μL of mixed template DNA, and 1 μL of a multiplex mix containing 10 μM primers and probes. The reaction protocol included an incubation at 50 °C for 2 min, pre-denaturation at 95 °C for 10 min, and 40 cycles of amplification at 95 °C for 15 s and 60 °C for 1 min.

### 3.3. Multiplex qPCR Standard Curves

**Figure 1:**
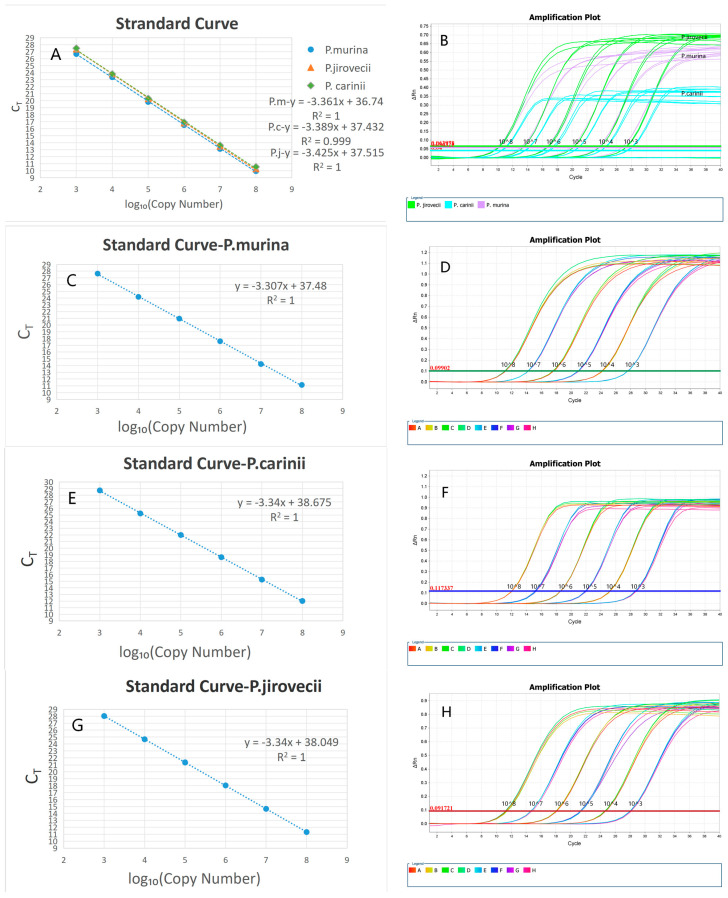
Standard curve (**A**) and amplification curve (**B**) of multiplex qPCR; standard curve (**C**) and amplification curve (**D**) of *P. murina* single-plex qPCR; standard curve (**E**) and amplification curve (**F**) of *P. carinii* single-plex qPCR; standard curve (**G**) and amplification curve (**H**) of *P. jirovecii* single-plex qPCR.

The plasmid standards pUC19-P.mMT, pUC19-P.cTS, and pUC19-P.jmt were serially diluted tenfold and subsequently combined in equal volumes at the same concentration to create a mixed template series. This preparation resulted in a final concentration for each plasmid that spanned six orders of magnitude, ranging from 1 × 10^8^ copies/μL to 1 × 10^3^ copies/μL. These templates were used for triplicate qPCR amplification to generate amplification curves and standard curves for each target gene. The equations derived from the standard curves were as follows: for *P. murina*, P.m-y = −3.361x + 36.74 (R^2^ = 1); for *P. carinii*, P.c-y = −3.389x + 37.432 (R^2^ = 0.999); and for *P. jirovecii*, P.j-y = −3.425 x + 37.515 (R^2^ = 1). The correlation coefficients (R^2^) for each equation exceeded 0.999, and the amplification efficiencies ranged from 90% to 110%. These results indicate that, within the concentration range of 1 × 10^8^ copies/μL to 1 × 10^3^ copies/μL, the copy numbers of the three recombinant plasmid standards exhibited strong linear relationships with the corresponding C_T_. Parallel single-plex qPCR standard curves, established under identical conditions, showed similarly excellent linearity (R^2^ ≥ 0.999) and amplification efficiencies (90–110%), with representative equations as follows: for *P. murina*, y = −3.307x + 37.48 (R^2^ = 1); for *P. carinii*, y = −3.34x + 38.675 (R^2^ = 1); for *P. jirovecii*, y = −3.34x + 38.049 (R^2^ = 1). No significant differences in slopes or C_T_ were observed between the multiplex and single-plex formats, confirming that multiplexing does not compromise quantitative performance (Figure 1).

### 3.4. Specificity Test Results

**Figure 2:**
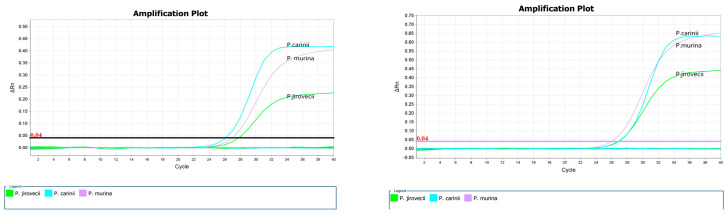
Specificity test results of multiplex qPCR. Only the positive control (DNA mix) produced amplification signals in the respective channels (VIC, CY5, FAM). No amplification was observed for any of the 22 non-target pathogens (10 rodent respiratory bacteria, 8 fungi, and 4 intestinal bacteria) or the negative control, confirming the high specificity of the assay.

Using the optimized multiplex qPCR system, 22 non-target pathogens (10 rodent respiratory bacteria, 8 fungi, and 4 intestinal bacteria) were tested as template DNA. No specific amplification curves or fluorescence signals were observed for any of the non-target pathogens in any channel (VIC for *P. murina*, CY5 for *P. carinii*, FAM for *P. jirovecii*). In contrast, only DNA mix (positive control) produced clear and distinct amplification signals in the corresponding channels, while the nuclease-free water negative control remained negative (Figure 2). These results demonstrate that the multiplex qPCR assay has excellent specificity for the simultaneous detection of *P. murina*, *P. carinii*, and *P. jirovecii*, with no cross-reactivity against the tested respiratory bacteria, partial fungal, or intestinal microorganisms commonly found in laboratory animals.

### 3.5. Sensitivity Test Results

**Figure 3:**
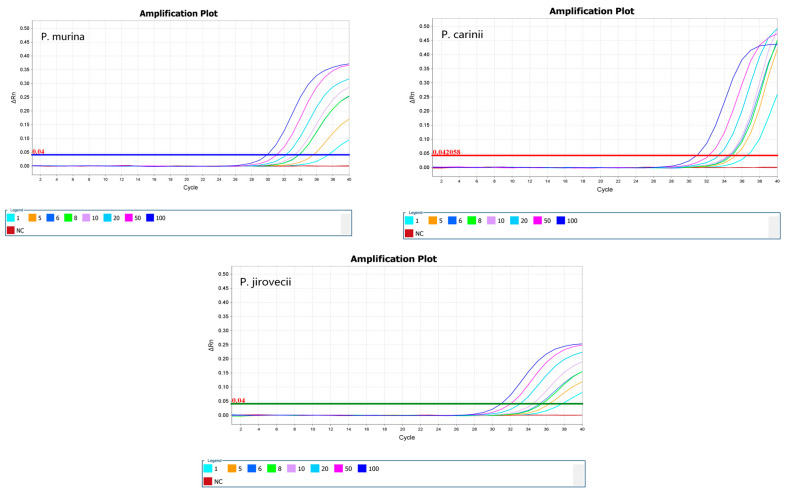
Sensitivity test results of multiplex qPCR for *P. murina*, *P. carinii*, and *P. jirovecii*.

The sensitivity test results indicated that the multiplex qPCR method exhibited high sensitivity for detecting the three *Pneumocystis* targets, with no amplification observed in the negative control (Figure 3). At plasmid concentrations of 100, 50, 20, 10, and 8 copies/μL, the detection rate for all three targets was consistently 100% (22/22). At lower concentrations (6, 5, and 1 copy/μL), the detection rates varied among the targets. The CV of C_T_ at each concentration was below 5%. Based on a detection rate threshold of ≥95%, the LODs of the multiplex qPCR method were determined to be 5 copies/μL for *P. murina*, 6 copies/μL for *P. carinii*, and 8 copies/μL for *P. jirovecii*. For comparison, single-plex qPCR assays performed in parallel at the same low concentrations (10, 8, 6, 5, and 1 copy/μL; 22 replicates each) showed LODs of 1 copy/μL for *P. murina*, 5 copies/μL for *P. carinii*, and 5 copies/μL for *P. jirovecii* under the same ≥95% criterion. These results confirm that multiplexing does not substantially compromise assay sensitivity. Detailed results for all low-concentration assays are presented in Table 3.

### 3.6. Repeatability Test Results

The reproducibility of the method was evaluated through intra-group and inter-group repeatability tests of the multiplex qPCR, using mixtures of the recombinant plasmid standards at concentrations of 1 × 10^3^ copies/μL, 1 × 10^5^ copies/μL, and 1 × 10^7^ copies/μL as templates. The results showed that the CVs for the intra-group repeatability test ranged from 0.08% to 0.78%, while the CVs for the inter-group repeatability test ranged from 0.17% to 0.69%. All CVs were below 1%, indicating that the assay demonstrates strong reproducibility (Table 4).

### 3.7. Spike-In Experiment Results

**Figure 4:**
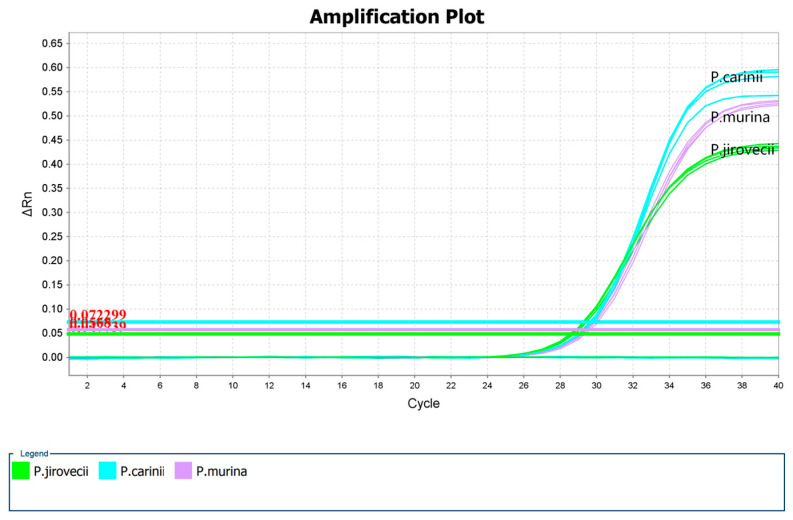
Amplification curves of the spike-in experiment. The curves for all experimental groups (including Groups E, F, and G) and all three *Pneumocystis* targets completely overlapped, indicating identical amplification kinetics across different matrix conditions and no detectable PCR inhibition. Negative controls (Groups H, I, J) showed no amplification.

Three independent spike-in experiments were performed to evaluate the extraction efficiency and qPCR inhibition for all three *Pneumocystis* targets. As shown in Figure 4, the amplification curves of Groups E, F, and G almost completely overlapped, indicating similar amplification kinetics across all conditions. The curves of Group E (matrix + plasmid after extraction) and Group F (plasmid only) were nearly identical, demonstrating no detectable PCR inhibition. Furthermore, the curves of Group G (extract from spiked matrix samples) also overlapped with those of Groups E and F, confirming that the extraction process did not compromise target recovery. All negative controls (Groups H, I, J) remained negative across all replicates. These results confirm that the sample processing protocol efficiently recovers target nucleic acids from lung tissue and BALF without significant inhibition or contamination.

### 3.8. Sample Detection Results

A total of 260 mouse lung samples and 30 *P. murina*-positive samples, 31 rat samples, and 8 *P. carinii*-positive samples were tested. multiplex qPCR and single-plex qPCR showed complete concordance (Table 5). Both methods detected 30 *P. murina*-positive samples and 8 *P. carinii*-positive samples, while all 260 mouse lung samples and 31 rat samples tested negative for *Pneumocystis* nucleic acid. For staining microscopy, Giemsa staining was performed on 68 mouse lung samples (all negative) and 38 positive samples (0 positive), while toluidine blue staining was performed on 5 positive samples (3 positive, detection rate 60.0%) (Table 6). The remaining samples were not examined by staining due to time constraints and sample limitation. The results obtained by Giemsa and toluidine blue staining were consistent with those of qPCR, but the positive detection rate of staining microscopy was markedly lower than that of qPCR (Table 7). The detection rate of the developed multiplex qPCR method was higher than that of staining microscopy.

### 3.9. Sequencing Verification Results

**Figure 5:**
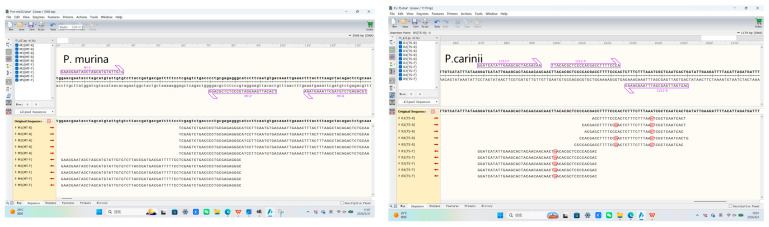
Sanger sequencing confirmation. Representative sequence alignments showing >95% identity of the amplified products to the reference sequences of *P. murina* (NC_020332.1 [2768–5511]) and *P. carinii* (XM_018371599.1).

Sanger sequencing confirmed the identity of the amplified products from *P. murina*-positive mouse samples and *P. carinii*-positive rat samples (Figure 5). The obtained sequences showed >95% identity to the respective reference sequences (GenBank accession numbers NC_020332.1 (2768–5511) for *P. murina* and XM_018371599.1 for *P. carinii*) over the entire amplicon length, with a few sequence mismatches observed [28]. Given that some degree of genetic variation is a normal phenomenon, these mismatches do not compromise the specificity of the designed primers nor the authenticity of the detected targets. The sequence of the *P. jirovecii* plasmid standard was not verified by sequencing as it was commercially synthesized with a documented sequence.

## 4. Discussion

In this study, a multiplex real-time fluorescence quantitative PCR (qPCR) method capable of simultaneously detecting and differentiating three common *Pneumocystis* species—*P. murina*, *P. carinii*, and *P. jirovecii*—was successfully established and preliminarily applied. This method provides a robust technical solution for the accurate and rapid detection of *Pneumocystis* infections specifically in laboratory animal quality control and related research settings.

Firstly, in terms of methodological development, the selection of target genes is critical for ensuring the specificity and sensitivity of the assay. For *P. murina*, the *mtLSUrRNA* gene was selected as the target. This gene exists in multiple copies in the genome, which significantly enhances detection sensitivity, and has been widely used for the molecular detection of animal-derived *Pneumocystis* [29,30]. For *P. carinii*, *TS* was selected as the target gene. Studies have shown that the *TS* gene exhibits species-specific polymorphisms among *Pneumocystis* species derived from different hosts, whereas its sequence is highly conserved within the same host species. This ensures the accurate identification of *P. carinii* and effectively avoids cross-reactivity with other *Pneumocystis* species or related microorganisms [31]. For *P. jirovecii*, the *mtSSUrRNA* gene was selected as the target. Phylogenetic studies have confirmed that this gene displays high interspecies polymorphism among *Pneumocystis* species from different host origins, with each mammalian host corresponding to a specific *mtSSUrRNA* sequence. Based on this property, the *mtSSUrRNA* gene effectively enables the specific recognition of human-derived *P. jirovecii* [32,33]. The optimized primer-probe combination achieved specific amplification of all three targets, and the cross-reactivity tests were negative.

In terms of detection performance, the detection limit of this system reached the single-copy level, meeting the requirements for detecting low-burden infections and subclinical states. The standard curve exhibited good linearity (R^2^ ≥ 0.999) and amplification efficiency (90–110%) within the range of 1 × 10^3^ copies/μL to 1 × 10^8^ copies/μL, ensuring accurate quantification and reliability over a wide dynamic range. Regarding sample processing, the use of grinding beads combined with low-temperature homogenization effectively disrupts cell walls and releases microbial nucleic acids. Subsequent extraction using a commercial kit minimized the inhibition of tissue impurities in the subsequent qPCR reaction, thereby laying the foundation for the stability and reproducibility of the assay.

Secondly, in the preliminary application validation, various samples—including lung tissues from uninfected and experimentally infected mice and rats, as well as rat BALF samples—were tested using the developed method. The results showed that the method stably detected the target pathogens and achieved strain differentiation based on the fluorescence signals in different channels. Among the mouse positive samples, the detection rate of *P. murina* was 100% (30/30); among the rat positive samples, the detection rate of *P. carinii* was 100% (8/8). The detection results for *P. jirovecii* in the corresponding samples were consistent with expectations (none detected), with no cross-contamination or non-specific amplification observed.

Compared with previously reported detection efficacies, a study on wild rodent populations reported an overall positive rate of *Pneumocystis* detection of 48.3% (215/445), with significant differences in infection rates among different rodent species [34]. A study on immunosuppressed laboratory rats reported a positive rate of *P. carinii* detection of 29.2% (7/24) by conventional etiological microscopy, whereas the positive rate by PCR in the same BALF samples was 91.7% (26/28), indicating a significantly higher detection efficacy of PCR over microscopic staining [35]. A study on commercial laboratory rat colonies reported that PCR without prior immunosuppression could detect latent *P. carinii* infection in 100% of PCR-positive lung samples, while histopathology after immunosuppressive induction was required for visual confirmation, with PCR showing superior sensitivity for routine monitoring [36]. Another study on BALF samples from immunocompromised patients showed that the positive rate of *P. jirovecii* detection using multiplex real-time qPCR was 40% (50/125), whereas the positive rate using conventional immunofluorescence was only 31.2% (39/125), again demonstrating that PCR outperforms fluorescence-based staining methods [37]. The detection rate of *P. murina* and *P. carinii* in the present study (100%) was higher than that reported for naturally infected populations described above. This may be attributed to the fact that the laboratory mice were maintained in a defined infection-exposed environment, as well as the high sensitivity conferred by the multicopy target gene (e.g., *mtLSUrRNA* for *P. murina*) used in this method, and to the ability of the optimized nucleic acid extraction process (grinding beads combined with low-temperature homogenization) to capture low-burden infections. Importantly, the present method achieved a 100% detection rate, surpassing not only the conventional staining/immunofluorescence-based approaches (which typically give much lower positivity, e.g., 29.2% for microscopy and 31.2% for immunofluorescence) but also the previously reported PCR results (e.g., 91.7% in rat BALF and 40% in human BALF). Therefore, the developed method shows advantages over traditional microscopy in terms of detection throughput, speed, and differential diagnostic capability for laboratory animal samples. It is particularly suitable for the simultaneous monitoring and precise prevention and control of *Pneumocystis* species derived from multiple host sources in laboratory animal facilities.

Although the present study has achieved preliminary results, certain limitations remain to be addressed in future work. It should be emphasized that the specificity, sensitivity, and reproducibility of the developed method have been systematically validated and demonstrated to be reliable: no cross-reactivity was observed with 22 common pathogens, the detection limit reached the single-copy level, and the coefficients of variation in both intra- and inter-group repeatability tests were below 1%, collectively confirming the accuracy and stability of the detection results [38,39]. With this as a foundation, the current validation included 290 mice (260 negative, 30 positive for *P. murina*) and 39 rats (31 negative, 8 positive for *P. carinii*). Compared with previously published studies, these sample sizes are within the range of those used for *Pneumocystis* detection assay development and validation, where reported numbers ranged from 24 to 445 animals depending on the study objectives [34,35,36,37]. For *P. jirovecii* infection samples, due to biosafety (BSL-2 requirements) [40,41] and ethical constraints (informed consent, IRB approval) [42], no authentic *P. jirovecii*-positive human samples were tested; validation for this target was performed using recombinant plasmid standards only. Future studies with clinical specimens are required before the assay can be applied to human PCP diagnosis. Furthermore, another limitation of this study is the lack of direct comparison with an established commercial or reference molecular assay for *Pneumocystis* detection. Currently, no commercially available multiplex kit is designed for simultaneous detection of *P. murina*, *P. carinii*, and *P. jirovecii* [43]. Future studies should include a comparative evaluation using well-validated single-plex qPCR assays or a reference laboratory-developed multiplex platform when available, to further confirm the relative performance of our assay.

Additionally, *Pneumocystis* species or genetic variants that have not yet been fully characterized may exist in the environment [44,45,46]. The ability of the method to identify potential variants should be continuously monitored in the future, and in cases where unintended amplification results are observed, sequencing should be performed for verification, thereby further confirming the specificity and robustness of the method.

Moreover, inter-laboratory comparisons and validations can be conducted through collaborative efforts to evaluate the applicability and consistency of the method under different operating environments, instrument platforms, and operators, thereby providing more comprehensive data support for its widespread application in laboratory animal quality control and scientific research practice [47].

## 5. Conclusions

In conclusion, the multiplex qPCR assay for *Pneumocystis* established in this study offers the advantages of high sensitivity, specificity, and throughput for detecting *Pneumocystis* infections in laboratory animals. It provides an efficient and reliable tool for etiological diagnosis, epidemiological investigations, and pathogenesis studies within laboratory animal settings. Future studies will focus on larger-scale validation studies involving multiple host sources to systematically evaluate the stability and applicability of this method across different animal populations. Based on these findings, a rapid diagnostic kit will be developed to promote the standardization and normalization of *Pneumocystis* detection technology. Additionally, by integrating artificial intelligence technology and combining detection data with multiple factors, such as host immune status and environmental conditions, a predictive model for *Pneumocystis* infection risk will be established, thereby providing decision-making support for the precise prevention and control of infections in laboratory animals. However, its application for human PCP diagnosis requires further clinical validation using authentic *P. jirovecii*-positive specimens, as the current study only performed analytical validation for this target.

## Figures and Tables

**Table 1:** Primer and probe sequence information.

Primers and Probes	Sequences (5′-3′)	Product Length/bp	Gene and Position
P.m-MT-F	GAACGAATACCTAGCATGTATTGTG	140	*mtLSUrRNA* NC_020332.1 (2768–5511)
P.m-MT-R	TGCAGAGTCTGTAGCTTAAAGTAAA		
P.m-MT-P	VIC-TCACATTGAAGGATGCCCTCTCGCAGG-BHQ1		
P.c-TS-F	GGATCATATTGAAGCACTACAACAA	83	*TS* XM018371599.1
P.c-TS-R	CAGTGATTGAGCGATTTAAAGAAAG		
P.c-TS-P	CY5-TTACACGCTCCCCACGACCTTTTCCCA-BHQ2		
P.j-mt-F	GACGTGCTGCAAAATTTTCTACAA	128	*mtSSUrRNA* NC_020331.1 (31,755–33,198)
P.j-mt-R	GACGATTACTAGCAATTCCAACTTC		
P.j-mt-P	FAM-AAGAGCCGAGTTCCAGGCACTTATCCG-BHQ1		

**Table 2:** Experimental design for spike-in assessment of extraction efficiency and qPCR inhibition.

Stage	Group	Template Composition	Spike-In (Copies/μL)	Purpose
DNA extraction	A	200 μL negative lung homogenate/BALF	None	Background control
	B	180 μL negative lung homogenate/BALF + 20 μL plasmid	10^3^	Extraction recovery monitoring
	C	180 μL sterile PBS + 20 μL plasmid	10^3^	Ideal recovery control
	D	200 μL nuclease-free water	None	Extraction blank
qPCR	E	2 μL extract from Group A + 1 μL plasmid	10^3^	Matrix inhibition assessment
	F	2 μL nuclease-free water + 1 μL plasmid	10^3^	Baseline control for inhibition
	G	2 μL extract from Groups B and C	None	Extraction recovery determination
	H	2 μL nuclease-free water	None	No-template control
	I	2 μL extract from Group A	None	Matrix negative control
	J	2 μL extract from Group D	None	Full-process blank control

**Table 3:** Sensitivity test results of multiplex and single-plex qPCR for three *Pneumocystis* species at low concentrations (1–10 copies/μL, n = 22).

Plasmid	Concentration (Copies/μL)	Multiplex qPCR Sensitivity Test				Single-Plex qPCR Sensitivity Test			
		Mean ± SD	CV (%)	Detection Rate (%)	95% CI	Mean ± SD	CV (%)	Detection Rate (%)	95% CI
P.m-MT	10	34.322 ± 0.441	1.283	100% (22/22)	(84.6%, 100%)	33.176 ± 0.715	2.155	100% (22/22)	(84.6%, 100%)
	8	34.611 ± 0.701	2.025	100% (22/22)	(84.6%, 100%)	33.465 ± 0.842	2.516	100% (22/22)	(84.6%, 100%)
	6	34.653 ± 0.359	1.036	100% (22/22)	(84.6%, 100%)	33.705 ± 0.641	1.902	100% (22/22)	(84.6%, 100%)
	5	35.150 ± 0.478	1.358	100% (22/22)	(84.6%, 100%)	33.916 ± 0.715	2.108	100% (22/22)	(84.6%, 100%)
	1	37.050 ± 0.803	2.167	50% (11/22)	(28.4%, 71.6%)	36.294 ± 0.854	2.353	95.5% (21/22)	(73.5%, 99.9%)
P.c-TS	10	33.052 ± 0.462	1.396	100% (22/22)	(84.6%, 100%)	34.443 ± 0.861	2.500	100% (22/22)	(84.6%, 100%)
	8	33.564 ± 0.670	1.995	100% (22/22)	(84.6%, 100%)	34.207 ± 0.740	2.163	100% (22/22)	(84.6%, 100%)
	6	33.994 ± 0.590	1.734	95.5% (21/22)	(73.5%, 99.9%)	34.660 ± 0.881	2.542	100% (22/22)	(84.6%, 100%)
	5	34.190 ± 0.734	2.147	86.7% (19/22)	(63.2%, 95.1%)	34.720 ± 0.854	2.460	100% (22/22)	(84.6%, 100%)
	1	35.823 ± 0.772	2.154	31.8% (7/22)	(16.0%, 55.2%)	35.176 ± 0.603	1.714	90.9% (20/22)	(68.5%, 96.5%)
P.j-mt	10	34.546 ± 0.543	1.570	100% (22/22)	(84.6%, 100%)	33.599 ± 0.550	1.637	100% (22/22)	(84.6%, 100%)
	8	34.484 ± 0.568	1.647	100% (22/22)	(84.6%, 100%)	34.084 ± 0.767	2.250	100% (22/22)	(84.6%, 100%)
	6	35.277 ± 0.627	1.777	90.9% (20/22)	(68.5%, 96.5%)	34.581 ± 0.696	2.013	100% (22/22)	(84.6%, 100%)
	5	35.392 ± 0.634	1.791	90.9% (20/22)	(68.5%. 96.5%)	34.370 ± 0.780	2.269	95.5% (21/22)	(73.5%, 99.9%)
	1	36.696 ± 0.835	2.275	63.6% (14/22)	(40.8%, 82.2%)	35.667 ± 0.665	1.864	50% (11/22)	(28.4%, 71.6%)

Note: Detection rate = positives/total (n = 22). 95% CI was determined by the Clopper–Pearson exact method (beta distribution).

**Table 4:** Intra-group and inter-group repeatability test results of multiplex qPCR.

Plasmid	Concentration (Copies/μL)	Intra-Group Repeatability Test		Inter-Group Repeatability Test	
		Mean ± SD	CV/%	Mean ± SD	CV/%
Pm-MT	1 × 10^3^	26.059 ± 0.046	0.18	26.142 ± 0.083	0.32
	1 × 10^5^	19.259 ± 0.050	0.26	19.337 ± 0.042	0.22
	1 × 10^7^	12.476 ± 0.051	0.41	12.620 ± 0.055	0.43
Pc-TS	1 × 10^3^	27.004 ± 0.022	0.08	27.172 ± 0.055	0.20
	1 × 10^5^	19.741 ± 0.051	0.26	20.082 ± 0.052	0.26
	1 × 10^7^	12.851 ± 0.068	0.53	13.223 ± 0.072	0.54
Pj-mt	1 × 10^3^	26.836 ± 0.031	0.12	26.734 ± 0.049	0.18
	1 × 10^5^	19.319 ± 0.041	0.21	19.513 ± 0.034	0.17
	1 × 10^7^	11.778 ± 0.092	0.78	12.505 ± 0.086	0.69

**Table 5:** Concordance analysis between multiplex qPCR and single-plex qPCR assays for *Pneumocystis* detection (N-mice = 290, N-rat = 39).

Multiplex qPCR Result	Single-Plex qPCR Result	No. of Samples	Concordance Rate
Mice-Positive	Mice-Positive	30	100%
Mice-Negative	Mice-Negative	260	100%
Rat-Positive	Rat-Positive	8	100%
Rat-Negative	Rat-Negative	31	100%

**Table 6:** Detailed detection rates of staining methods in a subset of *Pneumocystis*-positive mouse and rat lung samples.

Staining Method	Samples Tested (n)	Positive Detected (n)	Detection Rate (%)	Missed Detection Rate (%)
Giemsa	38	0	0.0	100.0
Toluidine Blue	5	3	60.0	40.0

**Table 7:** Comparative performance of multiplex qPCR, single-plex qPCR and staining microscopy for *Pneumocystis* detection in mouse and rat lung samples.

Sample Group	Total Samples (N)	Detection Method	No. of Samples Tested	Positive Detected (n)	Detection Rate (%)	Negative Detected (n)	Reason for Not Testing
Background Colony Mice	260	Multiplex qPCR	260	0	0.0	260	N/A
		Single-plex qPCR	260	0	0.0	260	N/A
		Giemsa Staining	68	0	0.0	68	Time constraints (n = 192)
		Toluidine Blue Staining	0	N/A	N/A	N/A	Not performed
*P.murina* Experimentally Infected Mice	30	Multiplex qPCR	30	30	100.0	0	N/A
		Single-plex qPCR	30	30	100.0	0	N/A
		Giemsa Staining	30	0	0.0	30	N/A
		Toluidine Blue Staining	5	3	60.0	2	Sample limitation (n = 25)
*P. carinii* Experimentally Infected Rat	8	Multiplex qPCR	8	8	100.0	0	N/A
		Single-plex qPCR	8	8	100.0	0	N/A
		Giemsa Staining	8	0	0.0	8	N/A
		Toluidine Blue Staining	0	N/A	N/A	N/A	Not performed

Abbreviation: N/A, not applicable.

## Data Availability

The original contributions presented in this study are included in the article. Further inquiries can be directed to the corresponding author.
